# School environment and mental health in early adolescence - a longitudinal study in Sweden (KUPOL)

**DOI:** 10.1186/s12888-016-0919-1

**Published:** 2016-07-16

**Authors:** Maria Rosaria Galanti, Hanna Hultin, Christina Dalman, Karin Engström, Laura Ferrer-Wreder, Yvonne Forsell, Martin Karlberg, Catharina Lavebratt, Cecilia Magnusson, Knut Sundell, Jia Zhou, Melody Almroth, Elena Raffetti

**Affiliations:** Department of Public Health Sciences, Centre for Epidemiology and Community Medicine (CES), Stockholm County’s Health Care District (SLSO), Karolinska Institutet, Widerströmska Huset, Tomtebodavägen 18a, 17177 Stockholm, Sweden; Department of Public Health Sciences, Karolinska Institutet, Stockholm, Sweden; Department of Psychology, Stockholm University, Stockholm, Sweden; Department of Education, Uppsala University, Stockholm, Sweden; Center for Molecular Medicine, Karolinska Institutet, University Hospital Sweden, Stockholm, Sweden; The Swedish Agency for Health Technology Assessment and Assessment of the Social Services, SBU, Stockholm, Sweden

**Keywords:** School environment, Mental health, Adolescence

## Abstract

**Background:**

Longitudinal studies indicate strong associations between school proficiency and indicators of mental health throughout adulthood, but the mechanisms of such associations are not fully elucidated. The Kupol study is a prospective cohort study in Sweden set up in order to: (i) describe the association of school pedagogic and social environment and its specific dimensions with the risk of mental ill-health and psychiatric disorders in adolescence; (ii) evaluate the direct effects of school pedagogic and social environment on mental health and the effects mediated by the individual’s academic achievements; and (iii) assess if school pedagogic and social environment are associated with mental ill-health through epigenetic mechanisms, in particular those involving genes regulating the response to stress.

**Methods:**

The Kupol cohort at baseline consists of 3959 children attending the 7th grade of compulsory school (13–14 years old) in 8 regions of central Sweden in the school years 2013–2014 or 2014–2015. Three follow-up surveys in subsequent years are planned. Teachers’ and students’ perceptions of the culture, climate and ethos of their schools, and students’ mental ill-health are assessed at the whole school level by annual questionnaire surveys. In order to conduct epigenetic analyses saliva specimens are collected from a nested sample of students at inception and two years later. Further, class-, family- and child-level information is collected at baseline and during each year of follow-up. Self-reported information is being complemented with register data via record-linkages to national and regional health and administrative registers.

**Discussion:**

The topic being investigated is new, and the sample constitutes the largest adolescent cohort in Sweden involved in an ad hoc study. Epigenetic analyses centered on environmental cues to stress response are a thoroughly new approach. Finally a notable feature is the multi-informant and multi-method data collection, with surveys at the school, class, family, and student level. Collaboration and data access: interested investigators should contact the coordinating centre. Additional information is available on the study’s website, http://kupolstudien.se/.

## Background

In Sweden, self-reported mental health problems and demand for psychiatric care among adolescents has increased in the last decades, and this is especially true for girls [1, 2]. Moreover, suicide rates have decreased in Sweden with the exception of the 10–24 age group, and suicide attempts have actually increased among girls in this age group [2].

Alongside the aforementioned trends in mental health problems, a gradual decrease in the literacy level of Swedish students has been noted for several years. According to the Programme for International Student Assessment (PISA), the ranking of Swedish students in the final year of compulsory school has been decreasing in all four assessments conducted between 2000 and 2010 [3].

Longitudinal studies indicate strong associations between school proficiency and indicators of mental health throughout adulthood, but the mechanisms of such associations are not fully elucidated [4–6]. In addition, the influence of school pedagogic and social environment as such on adolescents’ mental health is insufficiently investigated, particularly in Sweden. Because of the multiple pathways involved, a singular focus is sometimes placed on what children bring with them when they enter schools (eg., children’s social background) rather than on school environment or the complex interaction between multiple influences.

The Kupol study (Swedish acronym for “Knowledge on young people’s mental health and learning”) is a prospective cohort study in Sweden set up in order to investigate changes in adolescent’s mental health in relation to changes in school-level factors.

Specifically, the study aims to: (a) describe the association of school pedagogic and social environment and their specific dimensions (e.g. pedagogic leadership, teaching methods, relations in school) with the risk of mental ill-health and psychiatric disorders in adolescence; (b) evaluate the direct effects of school pedagogic and social environment on mental health and the effects mediated by the individual’s academic achievements; and (c) assess if school pedagogic and social environment are associated with mental ill-health through epigenetic mechanisms, in particular those involving genes regulating the response to stress.

Because of the extensive information accrued (described in detail below), the Kupol study allows for the exploration of highly relevant areas in addition to its primary objectives, such as: feasibility and effectiveness of screening for early detection of mental health problems among adolescents, costs of mental illness among school children, interaction between school and family-level psycho-social factors and mental ill-health, access to health-care interventions for sub-clinical mental health problems.

## Methods/Design

### Participants and recruitment of study population

The target for the cohort recruitment was constituted by children attending the 7th grade of compulsory school in both urban and rural areas of 8 regions of southern and central Sweden: Gävleborg, Jönköping, Stockholm, Södermanland, Uppsala, Värmland, Västmanland, and Örebro. The recruitment took place during the school years 2013–2014 and 2014–2015. In total, the target population in these regions was estimated to be of approximately 44000 students in each school year, representing about 44 % of the Swedish population in this age group.

#### Study population

##### Schools

**Fig. 1:**
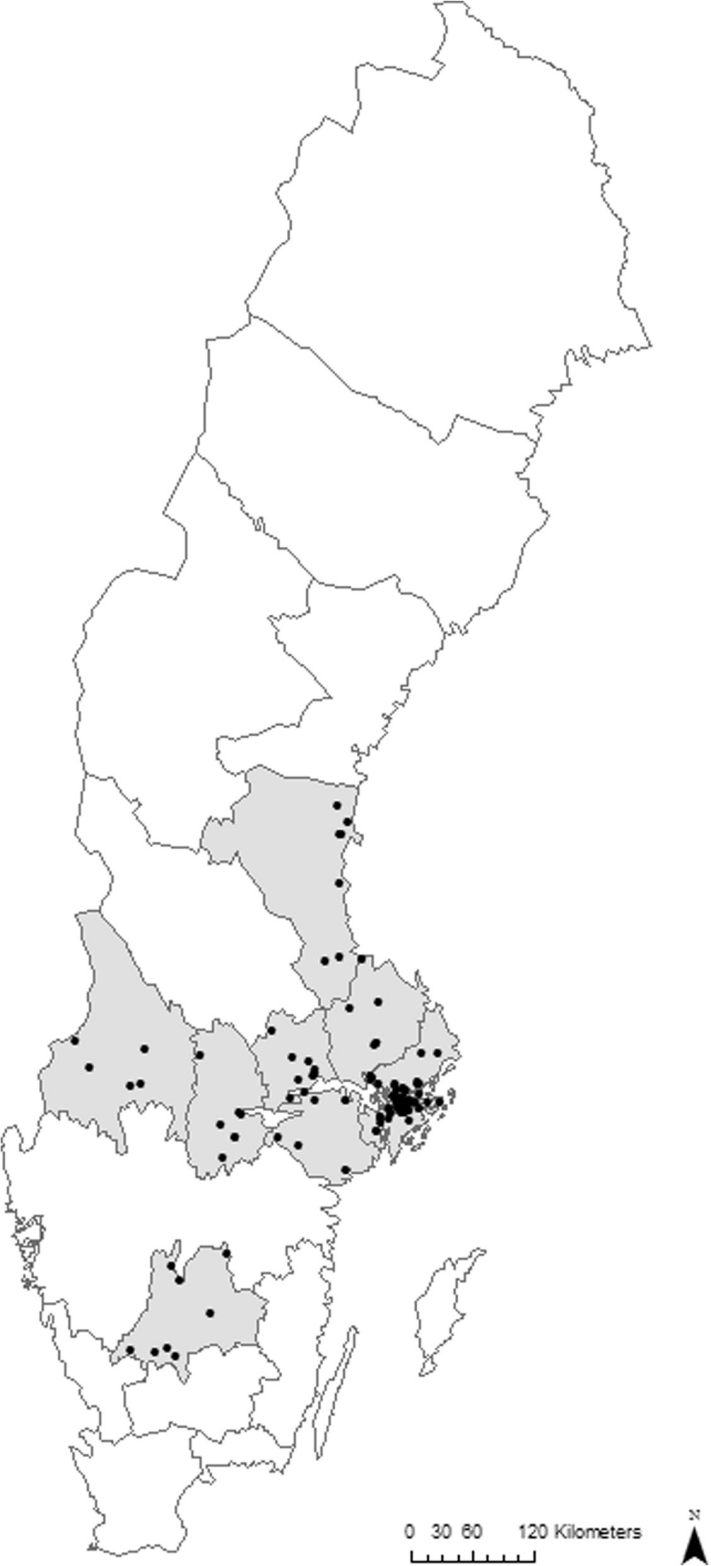
Geographic location of the schools participating in the Kupol Study, Sweden 2013–2015

The recruitment of schools in the Kupol study started during the spring semester of 2013. To be eligible, schools had to register at least 20 students per grade in the upper block (grade 7 to 9). In all, 541 schools were contacted, of which 6 were not eligible. Of the eligible schools, 101 (19 %) were willing to participate in the study while 434 (81 %) declined study participation. Figure 1 shows a map of the schools’ locations.

##### Parents and students

All students registered in the 7th grade in the two school years mentioned above were potentially eligible. Exclusion criteria included severe learning disabilities and/or poor comprehension of the Swedish language.

**Fig. 2:**
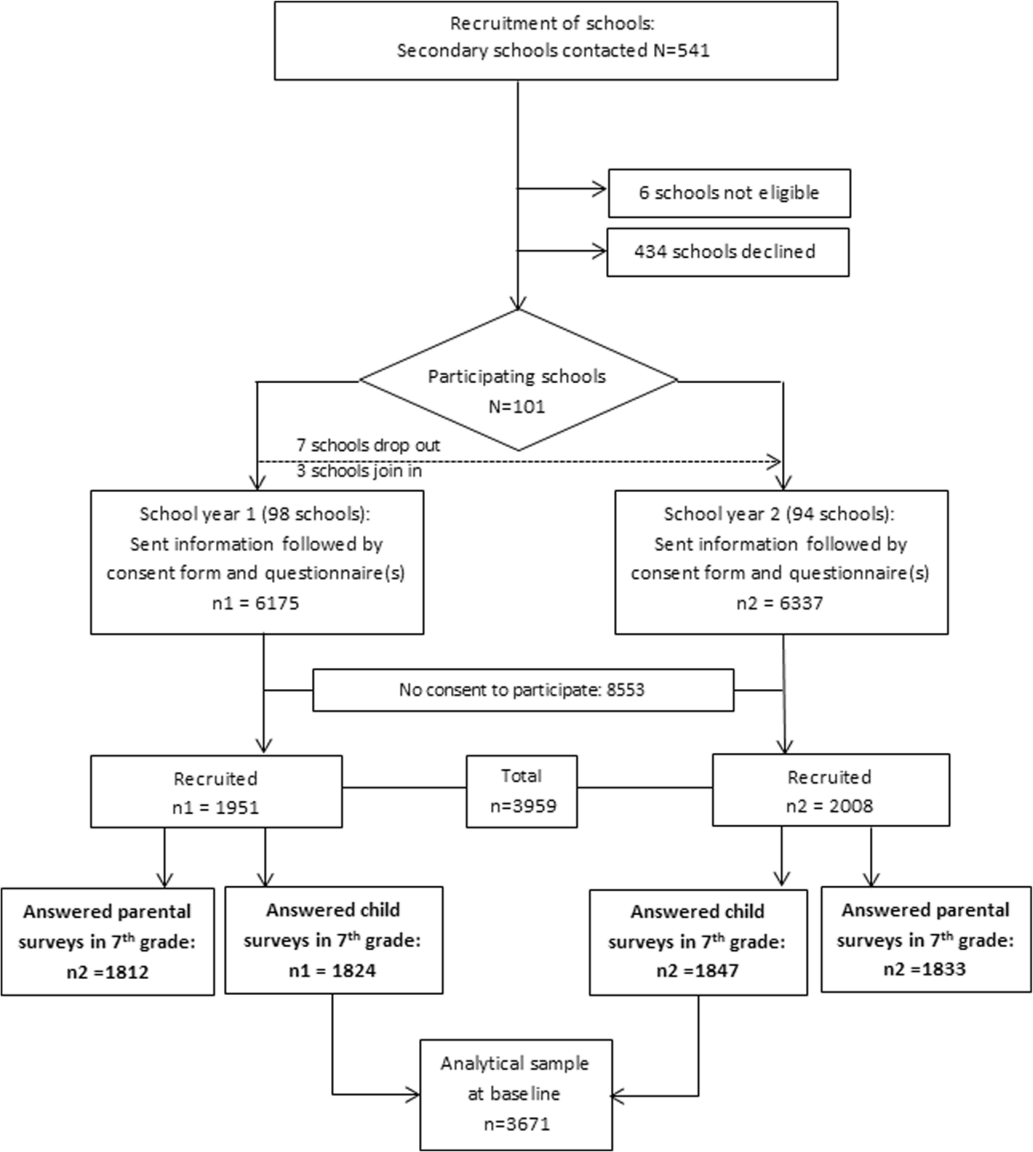
Derivation of the cohort in the Kupol Study, Sweden 2013–2015

An invitation to participate, including contact information to the project team, was forwarded by the school using the usual channels for school-family communication. The students also received a shortened version of the same information directly in schools. The initial contact was followed by mail delivery of written material with a complete description of the aim of the study, data collection methods and an explanation of the intended use of the collected data. A consent form and parent questionnaire was also sent to the adolescents’ guardians at the address of the legal residence where the adolescents were registered. Upon request, all material was made available into 10 different languages. Written informed consent to the child’s participation was required from parents/guardians, separately for the three components of data collection (survey, record linkage with registers, saliva sampling and epigenetic analyses). A flow-chart of the participants’ recruitment is displayed in Fig. 2.

In Table 1, participating and non-participating schools are compared regarding selected characteristics of the organization and of the student population. Notable differences concerned the authority responsible for running the school (participation among municipality schools was about half that among private school), and the average proportion of students with foreign born parents which was higher in the non-participating schools (25.5 %) than in the participating schools (19.4 %). Schools with a high proportion of teachers with a University degree were more represented among non-participating schools, but the mean difference was modest.

**Table 1:** Description of the participating and non-participating schools [n, mean ± standard deviation (SD) for continuous variables or n (%) for categorical variables]

Characteristic	Participating	Non-participating	*p*-Value
	*N* = 101	*N* = 434	
	N (%)	N (%)	
Responsible authority			0.0002
Public (Municipality)	62 (61.4)	342 (78.8)	
Private	39 (38.6)	92 (21.2)	
County			0.5347
Stockholm	51 (50.5)	234 (53.9)	
Other	50 (49.5)	200 (46.1)	
Average number of students per teacher (SD)	13.3 (2.7)	12.9 (2.4)	0.1914
Average proportion of teachers having University degree (SD)	79.1 (11.9)	81.6 (11.2)	0.0416
Proportion of teachers with University degree			0.3767
High (>87)	28 (28.0)	145 (34.7)	
Medium (78.5–87)	34 (34.0)	139 (33.3)	
Low (<78.5)	38 (38.0)	134 (32.1)	
Average proficiency score of students in the 9th grade in 2014 (SD)	221.8 (25.4)	217.3 (26.3)	0.1261
Mean number of students in school (SD)	371.8 (185.5)	399.0 (196.6)	0.2106
Number of students in school			0.7643
High (>432)	31 (31.3)	145 (33.7)	
Medium (279–432)	32 (32.3)	145 (33.7)	
Low (<279)	36 (36.4)	140 (32.6)	
Average number of students per grade (SD)	64.2 (34.7)	69.9 (38.4)	0.1708
Average number of students per grade			
High (>78.4)	32 (32.3)	145 (33.7)	0.8875
Medium (47.1 – 78.4)	32 (32.3)	144 (33.5)	
Low (>47.1)	35 (35.4)	141 (32.8)	
Average proportion of students with foreign background (SD)	19.4 (16.5)	25.5 (22.0)	0.0031
Proportion of students with foreign background, tertiles			0.0383
High (>24)	22 (23.9)	143 (35.0)	
Medium (12–24)	31 (33.7)	144 (35.2)	
Low (<12)	39 (42.4)	122 (29.8)	
Average proportion of students with parents with university education, (SD)	55.2 (16.5)	52.6 (17.2)	0.1879
Proportion of students with parents with university education, tertiles			0.3580
High (>61)	33 (34.0)	137 (32.3)	
Medium (44–61)	37 (38.1)	138 (32.6)	
Low (<44)	27 (27.9)	149 (35.1)	

In Table 2, the odds ratios of school participation are presented separately for privately and publically run schools according to selected school characteristics. Increasing numbers of students in each grade predicted lower participation among private schools, while increasing proportion of students born outside Sweden or with non-Swedish parents was associated with lower school participation in both privately and publically run schools.

**Table 2:** Odds Ratios (ORs) and 95 % confidence intervals of participation among private and public schools according to selected school characteristics, Kupol study, Sweden 2013

Characteristic	Private school (*N* = 131)	Public school (*N* = 404)	All schools (*N* = 535)
County			
Stockholm	1	1	1
Other	1.02 (0.45, 2.29)	1.57 (0.90, 2.72)	1.15 (0.74, 1.77)
Number of students per teacher	1.04 (0.89, 1.21)	1.01 (0.90, 1.13)	1.06 (0.97, 1.15)
Percentage of teachers with University degree	0.995 (0.970, 1.021)	1.001 (0.969, 1.034)	0.981 (0.964, 0.999)*
Proficiency score for students in the 9th grade in 2014	1.004 (0.987, 1.022)	0.998 (0.986, 1.009)	1.007 (0.998, 1.015)
Number of students at school	0.998 (0.996, 1.001)	1.000 (0.999, 1.001)	0.999 (0.998, 1.000)
Average number of students per grade	0.986 (0.974, 0.999)*	1.003 (0.996, 1.010)	0.996 (0.990, 1.002)
Percentage of students with foreign background	0.970 (0.942, 0.999)*	0.988 (0.973, 1.003)	0.983 (0970, 0.997)*
Percentage of students with parents with university education	1.007 (0.979, 1.035)	0.995 (0.978, 1.013)	1.009 (0.996, 1.022)
Percentage of teachers with University degree			
High (>87)	0.39 (0.10, 1.48)	1.11 (0.55, 2.23)	0.68 (0.40, 1.17)
Medium (78.5–87)	1.67 (0.67, 4.14)	1.02 (0.50, 2.07)	0.86 (0.51, 1.45)
Low (<78.5)	1	1	1
Number of students at school			
High (>432)	0.78 (0.31, 1.94)	1.11 (0.55, 2.23)	0.83 (0.49, 1.42)
Medium (279–432)	0.53 (0.19, 1.42)	1.33 (0.67, 2.62)	0.86 (0.51, 1.46)
Low (<279)	1	1	1
Number of students at school per grade			
High (>78.4)	0.24 (0.06, 0.87)*	1.80 (0.89, 3.65)	0.89 (0.52, 1.51)
Medium (47.1–78.4)	1.29 (0.53, 3.13)	1.18 (0.56, 2.49)	0.90 (0.53, 1.53)
Low (<47.1)	1	1	1
Percentage of parents with foreign background			
High (>24)	0.54 (0.19, 1.52)	0.45 (0.22, 0.91)*	0.48 (0.27, 0.86)*
Medium (12–24)	0.96 (0.36, 2.54)	0.59 (0.30, 1.10)	0.67 (0.40, 1.14)
Low (<12)	1	1	1
Percentage of parents with higher education			
High (>61)	0.57 (0.24, 1.36)	0.67 (0.31, 1.45)	0.90 (0.53, 1.52)
Medium (44–61)	1	1	1
Low (<44)	-	0.96 (0.52, 1.75)	0.68 (0.39, 1.17)

*p < 0.05

A total of 3959 school children agreed to participate, of which 3671 (48.2 % boys) provided response to baseline (92.7 %). Table 3 reports the socio-demographic characteristics of the children’s families. The large majority of participants lived in families with highly educated parents, with at least one parent employed and/or born in Sweden.

**Table 3:** Family’s socio-demographic characteristics, the Kupol Study, Sweden 2013–2014

		N (%)
Parental highest achieved education	University/College	2308 (68.3)
	Secondary school	1001 (29.6)
	Primary school or no schooling	72 (2.1)
	Missing	2
Parental employment	At least one parent employed	3291 (97.5)
	Neither parent employed	85 (2.5)
	Missing	7
Geographical origin of parents	At least one parent born in Sweden	3022 (90.8)
	Neither parent born in Sweden	308 (9.2)
	Missing	53
Index child’s living arrangement	With both parents	3419 (93.2)
	With one parent only	235 (6.4)
	With none of the parents	14 (0.4)
	Missing	3

#### Follow-up

**Fig. 3:**
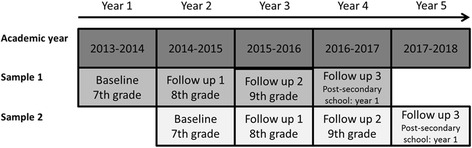
Timeline of data collection in the cohort sub-samples, The Kupol Study, Sweden

In total, three follow-up surveys in subsequent years are planned for each sub-sample in the cohort (i.e. children recruited in the school years 2013–2014 or 2014–2015). Two of the surveys are conducted while the children are still in the compulsory school, while the third one will be conducted during the years of the transition to upper secondary school or equivalent (2017–2019). Figure 3 shows the timeline for data collection.

To date, baseline data have been collected for the whole cohort, while follow-up data have been collected for the sub-cohort recruited in 2013–2014 (proportion retained 97.7 %). Multi-informant and multi-method data collection is employed, with surveys at the school, classroom, family, and student level, using both paper and web-based instruments. Self-reported information is complemented with register data via record-linkages to national and regional health and administrative registers (Table 5).

#### Information collected

Table 5 provides a detailed description of the information collected in the study. The paragraph below provides an overview of the main information and of data collection procedures among school personnel, participating students and their parents.

##### School pedagogic and social climate

Teacher and student perceptions of the culture, climate and ethos of their schools are assessed using the PESOC scale (Pedagogical and Social Climate of a school, http://gauss.stat.su.se/rr/RR2004_6.pdf), an instrument available as a teacher or student version. The instrument for teachers consists of a self-completed questionnaire containing 67 items, while the corresponding instrument for students contains 53 items. In each school, the instrument is administered to all teaching personnel and to all students attending the 9th grade, i.e. the last compulsory school year.

Respondents are asked to rate their level of agreement with the statements on a 4 point-Likert scale ranging from “not at all” to “completely agree”. A fifth option for “don’t know” is also included. Resting on theoretical principles, the statements are grouped to form subscales assessing different domains (e.g. for students “perceived school expectations” or “perceptions of teachers’ support”; for teachers “pedagogic leadership of the principal” or “cooperation among teachers”). There are eight such domains in the student instrument and eleven in the teacher instrument. A comprehensive psychometric analysis of the PESOC instruments is under publication [7, 8].

##### Strengths and Difficulties Questionnaire and Center for Epidemiologic Studies Depression Scale for Children

Mental ill-health is primarily assessed by means of multi-informant (parent, child) completion of the 25-item Strengths and Difficulties Questionnaire (SDQ). The SDQ evaluates five domains: hyperactivity-inattention, emotional symptoms, conduct problems, peer problems and prosocial behaviour [9]. The SDQ is included in the parents’ and children’s questionnaire at baseline and each subsequent follow-up. In order to increase the sensitivity of the mental ill-health assessment in the domain of internalizing problems, the Center for Epidemiologic Studies Depression Scale for Children (CES-DC), a 20-item scale is also included in the adolescent questionnaire [10].

Indicators of general and mental health among participant children at baseline are shown in Table 4, separately by gender.

**Table 4:** Self-reported general and mental health among participant children at baseline, the Kupol Study, Sweden 2013–2014

		All	Girls	Boys	*p*-Value
		(*N* = 3671)	(*n* = 1901)	(*n* = 1770)	
General health					
	Very good	2171 (59.9)	1019 (54.5)	1152 (65.8)	<.0001
	Fairly good	1360 (37.6)	792 (42.3)	568 (32.5)	
	Not very good	90 (2.5)	60 (3.2)	30 (1.7)	
	Missing	50	-	-	
Mental health					
Parent-reported SDQ^a^	Indicates problem (17–40)	154 (4.6)	75 (4.3)	79 (4.8)	0.0529
	Borderline (14–16)	125 (3.7)	52 (3.0)	73 (4.5)	
	Doesn’t indicate problems (0–13)	3090 (91.7)	1608 (92.7)	1482 (90.7)	
	Unscored	302	-	-	
Child-reported SDQ	Indicates problem (20–40)	183 (5.0)	135 (7.2)	48 (2.7)	<.0001
	Borderline (16–19)	350 (9.7)	209 (11.1)	141 (8.1)	
	Doesn’t indicate problems (0–15)	3092 (85.3)	1538 (81.7)	1554 (89.2)	
	Unscored	46	-	-	
CES-DC	Indicates problem (≥30)	346 (9.6)	297 (15.9)	49 (2.8)	<.0001
	Doesn’t indicate problems (<30)	3265 (90.4)	1577 (84.1)	1688 (97.2)	
	Unscored	60	-	-	

*P*-Value refers to *X* ^*2*^ test for categorical variables in gender comparison

^a^Available for 3662 participants

At baseline, most children reported good or very good general health, with only 2.5 % reporting poor health. Scale scores indicating mental health problems were found among 5 % of the participants using the child SDQ and among 9.6 % using the CES-DC. Consistent differences in self-reported health were observed between genders, with girls reporting very good general health to a lesser extent than boys (54.5 % vs 65.8 %). Scale scores indicating mental health problems were also more frequent among girls, in both SDQ and CES-DC. Such differences between genders were not present in parent-reported SDQ, despite the overall estimate of the prevalence of children with high scores (4.6 %) was close to the children-based assessment. In other words, average parents’ rating seemed to overestimate the frequency of boys’ mental health problems compared to the children’s self-ratings, while the opposite was true for girls.

##### Biological samples and study of epigenetics

Saliva samples are collected from a nested random sample of about 1500 consenting students at baseline (7th grade) and again at the end of the 9th grade. On each occasion, three saliva samples are collected from each participant, two of which will be used to determine morning and afternoon cortisol levels, while the third sample will be used for genetic and epigenetic analyses, in particular the methylation of deoxyribonucleic acid (DNA) in regulatory regions associated to mental ill-health and/or to childhood/adolescence adversities. Such epigenetic modifications have particular importance because they can influence the developing person through-out the lifespan, providing evidence that environmental stress can be cemented in the genome through DNA methylation [11]. All samples are stored in the Karolinska University Hospital Biobank.

##### Other socio-demographic and psycho-social factors

In order to have insight into potential confounding factors, as well as to study mediatory and interaction effects, further information about class, family, and child characteristics has been collected at baseline and at follow-up.

Items at the classroom-level are included in a teacher questionnaire, asking about classroom composition, formal competence of the teachers in the core subjects, curricular activities for each core subject, cross-disciplinary, special pedagogic activities, social climate in the classroom, i.e. perception of discipline/absenteeism, the quality of relations among students, between students and teachers, and among teachers.

The parental questionnaire includes items on composition of the family and the index child’s cohabitation forms, parental employment, attitudes and expectations towards school achievements, bonding within family, and own use of alcohol and tobacco.

The child questionnaire includes items on bonding to parents and peers, lifetime and recent use of tobacco, alcohol and illicit drugs, height and weight, self-rated health, and relation to school.

##### Register-based information

In order to refine the assessment of mental health outcomes, information on clinical diagnoses will be accrued through record-linkages with national or regional health registers, such as the Hospital Discharge Register, the Swedish Prescribed Drug Register, and the child neuropsychiatric outpatient clinics (BUP) (Table 5). Other information accrued from health or administrative registers include school absenteeism (school computerized records) and school grades (The Swedish National Agency of Education); socio-economic factors such as parental income and parental sickness and disability benefit (The Longitudinal Integration Database for Health Insurance and Labour Market Studies, LISA). Table 5 gives an overview of these record-linkages, which are deterministic because they are based on a unique individual identifier (personal registration number, PNR) assigned to all residents in Sweden at birth or at the time of entering the country.

**Table 5:** Summary of the main information collected in the Kupol study at baseline and follow-up

Information	Source	Instrument
School infrastructure and organization	School principal	Paper questionnaire
School pedagogic and social climate	Teachers and students attending the last year of the compulsory school	Paper and/or web-based questionnaire
Classroom composition and teaching style	Teachers in math, Swedish and English	Paper questionnaire
Family demographics (parental education, employment, geographic origin)	Parents/guardians	Paper questionnaire
Parents’ socioeconomic factors (education, income, occupation, sickness and disability benefits)	LISA register	Record linkage
Parents’ perceived own health, tobacco and alcohol use	Parents/guardians	Paper questionnaire
Parents’ social capital	Parents/guardians	Paper questionnaire
Parents’ relation to the index child	Parents/guardians	Paper questionnaire
Parents’ expectations on index child’s academic achievements	Parents/guardians	Paper questionnaire
Child’s peri-natal events and anthropometric characteristics	Medical Birth Register	Record linkage
Child’s mental health (SDQ)	Parents/guardians and index child	Paper questionnaire
Child’s mental health (CES-DC)	Index child	Paper questionnaire
Child’s psychiatric diagnoses	Inpatient and outpatient registers	Record linkage
Child’s pharmacologic treatments	Swedish Prescribed Drug Register	Record linkage
Substance use	Index child	Paper questionnaire
Perceived general health	Index child	Paper questionnaire
Relation to parents	Index child	Paper questionnaire
Relation to school and academic expectations	Index child	Paper questionnaire
School absenteeism	School registers	Record linkage
Relation to peers	Index child	Paper questionnaire
Experience of bullying	Index child	Paper questionnaire

#### Sample size and statistical power

The accrued sample will provide more than 80 % power to detect as statistically significant (alpha = 0.05) a 50 % increase in the risk of mental health problems among students exposed to the lowest quartile of PESOC score compared to the higher quartiles assuming a cumulative incidence among the unexposed as low as 8 %, taking into account the cluster design.

#### Data/statistical analysis

Because the accrued data will have an inherent hierarchical structure, with students grouped in classes and schools, multilevel regression modeling will be the elective analytical method. Both the primary outcome (SDQ-based mental health problems) and most secondary outcomes (e.g. substance abuse, conduct disorders, internalizing symptoms) will be primarily categorized as dichotomous. Therefore, we will use logistic regression to model yearly and cumulative incidence. The primary exposure (PESOC) will be analyzed as a continuous as well as a categorical variable. In the main analysis we will explore the mediatory and effect-modifying role of school proficiency, other individual-level factors (above all, gender) and out-of-school factors (e.g. family circumstances) on the association between overall school pedagogic climate, its sub-dimensions and occurrence of mental disorders. Potential confounders to be adjusted for mainly consist of familial social circumstances such as migration status and social adversity, which may indeed impact on choice of school as well as mental health. Given the complex interplay of causal and non-causal relations occurring between different levels of environmental and individual factors the primary analyses will be complemented with alternative estimation and testing techniques, such as structural equation modeling.

Finally, conditional logistic regression will be used to analyze the case-control study of DNA methylation and cortisol levels.

## Discussion

The main strengths of the Kupol study include its longitudinal design, with yearly data collection during and beyond upper primary school, and the wide range of information available on different levels, using multi-informant and multi-method assessments. A rich data set is being compiled through the use of registers, surveys at the school, classroom, student and parent level, as well as through collection of biological samples.

The low baseline response rate at the school (19 %) and student (29 %) levels may have contributed to greater than intended inclusion of high socio-economic status participants. The total sample at inception of 3959 adolescents makes this the largest ad hoc cohort of adolescents in Sweden, i.e. with collection of original data. Further, the follow-up rate was very good. There was a high retention rate (97.7 %) from baseline to the first follow-up for the earliest included sample. In terms of expertise, the Kupol study brings together newly-trained and well-established researchers across a wide range of disciplines (medicine, epidemiology, social sciences, education, and psychology) who share an interest in young people’s mental health. In summary, the Kupol study cohort provides a unique platform to evaluate the association between different dimensions of school level factors and mental health among adolescents.

Interested investigators should contact the corresponding author at the coordinating centre. More information can be found on the study’s website, http://kupolstudien.se/.

## Abbreviations

CES-DC, Center for Epidemiologic Studies Depression Scale for Children; DNA, Deoxyribonucleic Acid; LISA, Longitudinal Integration Database for Health Insurance and Labour Market Studies; PESOC, Pedagogical and Social Climate of a school; PISA, Programme for International Student Assessment; PNR, Personal Registration Number; SDQ, Strengths and Difficulties Questionnaire

## References

[1] Folkhälsomyndigheten. Folkhälsan i Sverige årsrapport 2014. Sweden: 2014

[2] Sciences TRSAo. Trends in Child and AdolescentMental Health in Sweden2010. Available from: http://www.buph.se/download/18.215ade6b1325af7d93380003585/1366677798136/RSAS_Healthcommitee_Statement_Trends_lowresolution.pdf. Accessed 4 July 2016.

[3] OECD. Improving Schools in Sweden: An OECD Perspective. Organisation for Economic Cooperation and Development 2015. Available from: http://www.oecd.org/edu/school/Improving-Schools-in-Sweden.pdf. Accessed 4 July 2016.

[4] Kosidou KDC, Fredlund P, Leea BK, Galantia R, Isacssona G, Magnussona C (2014). School performance and the risk of suicide attempts in young adults: a longitudinal population-based study. Psychological Medicine.

[5] Chong SASM, Lee IM, Pek E, Cheok C, Verma S, Wong J (2009). Academic attainment: a predictor of psychiatric disorders?. Social Psychiatry and Psychiatric Epidemiology..

[6] Verboom CE, Sijtsema JJ, Verhulst FC, Penninx BW, Ormel J (2014). Longitudinal associations between depressive problems, academic performance, and social functioning in adolescent boys and girls. Developmental Psychology.

[7] Hultin H, Ferrer-Wreder L, Dimitrova R, Karlberg M, Galanti MR. Psychometric Properties of an Instrument to measure Social and Pedagogical School Climate among Teachers (PESOC). (Accepted, Scandinavian Journal of Educational Research)

[8] Hultin H, Ferrer-Wreder L, Dimitrova R, Karlberg M, Galanti MR.Pedagogical and Social School Climate: Psychometric Evaluation and Validation of the Student Edition of PESOC. (Manuscript submitted )

[9] Goodman R, Ford T, Simmons H, Gatward R, Meltzer H (2000). Using the strengths and difficulties questionnaire (SDQ) to screen for child psychiatric disordersin a community sample. British Journal of Psychiatry..

[10] Fendrich M, Weissman MM, Warner V (1990). Screening for depressive disorder in children and adolescents: validating the Center for Epidemiologic Studies Depression Scale for Children. Am J Epidemiol.

[11] McGowan PO, Szyf M (2010). The epigenetics of social adversity in early life: implications for mental health outcomes. Neurobiology of disease.

